# A High-Sensitivity Microfluidic Sensor Based on a Substrate Integrated Waveguide Re-Entrant Cavity for Complex Permittivity Measurement of Liquids

**DOI:** 10.3390/s18114005

**Published:** 2018-11-16

**Authors:** Zhihua Wei, Jie Huang, Jing Li, Guoqing Xu, Zongde Ju, Xuyang Liu, Xingsheng Ni

**Affiliations:** College of Engineering and Technology, Southwest University, Chongqing 400715, China; wzh983700424@email.swu.edu.cn (Z.W.); lj940319@email.swu.edu.cn (J.L.); guoqingxu101@email.swu.edu.cn (G.X.); fghj2016@email.swu.edu.cn (Z.J.); lxy1998@email.swu.edu.cn (X.L.); n740689257@email.swu.edu.cn (X.N.)

**Keywords:** microwave sensor, microfluidics, SIW re-entrant cavity resonator, complex permittivity measurement

## Abstract

In this study, a novel non-invasive and contactless microwave sensor using a square substrate integrated waveguide (SIW) re-entrant cavity is proposed for complex permittivity measurement of chemical solutions. The working principle of this sensor is based on cavity perturbation technique, in which the resonant properties of cavity are utilized as signatures to extract the dielectric information of liquid under test (LUT). A winding microfluidic channel is designed and embedded in the gap region of the cavity to obtain a strong interaction between the induced electric field and LUT, thus achieving a high sensitivity. Also, a mathematical predictive model which quantitatively associates the resonant properties of the sensor with the dielectric constant of LUT is developed through numerical analysis. Using this predictive model, quick and accurate extraction of the complex permittivity of LUT can be easily realized. The performance of this sensor is then experimentally validated by four pure chemicals (hexane, ethyl acetate, DMSO and water) together with a set of acetone/water mixtures in various concentrations. Experimental results demonstrate that the designed sensor is capable of characterizing the complex permittivities of various liquids with an accuracy of higher than 96.76% (compared with the theoretical values obtained by Debye relaxation equations), and it is also available for quantifying the concentration ratio of a given binary mixture.

## 1. Introduction

Over the past two decades, accurate complex permittivity measurement of materials/liquids in the microwave region has aroused considerable enthusiasm as it shows great potential in many areas such as biomedical, chemical, agriculture and food grading [1,2,3,4]. Researchers hitherto have developed many methods for characterizing complex permittivity, which can be grossly divided into two categories: transmission methods and resonant methods. Compared to transmission methods, the resonant ones are preferable due to their higher accuracy, most notably for characterization of low-loss materials. Thus, a variety of resonators have been proposed and utilized for material characterization, including split-ring resonators (SRRs) [5,6,7], cavity resonators [8,9], metamaterial-based resonators [10,11], half/quarter wavelength resonators [12,13] and dielectric resonators [14]. Among these resonators, the re-entrant cavities have attracted significant interest since they offer the advantages of high Q-factor and highly concentrated electromagnetic fields, with the result that a number of complex permittivity measurement concepts based on the re-entrant cavities are reported [15,16,17]. However, the traditional re-entrant cavity resonators generally suffer from bulky volumes and high costs, and their non-planar structures also make them difficult to be integrated with other planar circuits (e.g.**,** data readout and postprocessing circuit). These drawbacks severely hinder their practical applications, especially in the case where a high-density integration is required.

Substrate integrated waveguide (SIW) technology, realized through rows of metallic via-hole arrays or grooves embedded in a dielectric substrate whose upper and lower surfaces are covered by metal plates, has attracted increasing attention in the last few years [18,19,20]. This technology makes it possible to design the re-entrant cavities in planar forms that can be readily fabricated using standard printed circuit board (PCB) or low-temperature co-fired ceramic (LTCC) technology. The SIW re-entrant cavities not only inherit the advantages of traditional cavities such as high Q-factor and high power capacity, but also provide the merits of low cost, low profile and easy fabrication. More importantly, their planar configurations make them much easier to be monolithically integrated with other planar circuits and systems (without any other costly and sophisticated transition [21]). Recently the SIW re-entrant cavities have been successfully applied to the fields of filter design [22,23,24], in which their excellent resonant performances and design simplicities have been well demonstrated. However, to the best of authors’ knowledge, little attention has been paid to their applications for liquid complex permittivity measurement.

In order to realize the interaction between the liquid and the induced electric fields and then the extraction of liquid dielectric properties, resonators are usually immersed in a mass of liquid specimens [13] or loaded by a micro capillary filled with samples [25]. Such methods, however, suffer from wastefulness of specimens or poor sensitivity. Fortunately, the emerging microfluidic technology provides an effective solution for these problems. By using pre-shaped channels with micrometer- or submillimeter-scale dimensions, microfluidics is able to precisely manipulate very small quantities of fluids, thus allowing a sensitive and accurate fluid analysis without any waste of samples. Moreover, it also has the merits of high resolution, small footprints and real time detections [26]. These features make the microfluidics a promising candidate for liquid dielectric properties analysis.

In this paper, a microfluidic-integrated square SIW re-entrant cavity with high Q-factor is proposed as a sensor to evaluate the dielectric property of liquids. The fluidic channel is designed in a winding shape and is embedded in the gap region of the re-entrant cavity, where a uniform electric field with extremely high intensity is excited. When a liquid sample is injected into the microfluidics, it would interact with the intense electric field, leading to the changes in resonance frequency and Q-factor (or 3-dB bandwidth) of the cavity. The complex dielectric constant of the sample can then be extracted from these changes. Compared to other recently reported sensors, the proposed one in our work shows distinct advantages such as compact size, high sensitivity and accuracy. In addition, it is also compatible with lab-on-a-substrate approach thanks to the utilization of SIW technology.

## 2. Working Principle

### 2.1. Cavity Perturbation Theory

**Figure 1:**
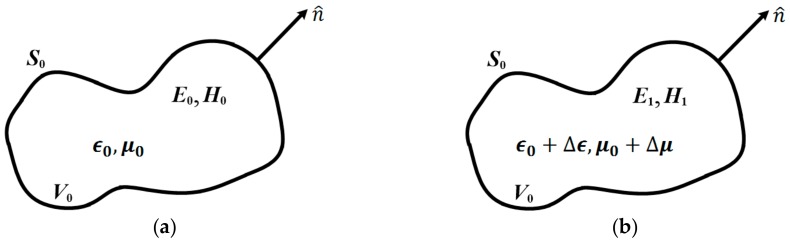
Diagram of the cavity perturbation: (**a**) empty cavity; (**b**) perturbed cavity.

The operating principle of the designed sensor is based on the well-known cavity perturbation approach. In this approach, the liquid under test (LUT) is introduced into the cavity and placed at the region where electromagnetic field is highly concentrated. The field distributions would then be perturbed due to the existence of LUT, thereby causing a significant redshift in resonance frequency and decrease in Q-factor. Figure 1 shows the diagram of the perturbation of a cavity. The resonance frequency and Q-factor associating with the dielectric properties of LUT are defined as [27,28]:

(1) $$\frac{f_{r}-f_{0}}{f_{0}}=\frac{-\int_{V_{0}}^{\;}\left(\Delta \epsilon E_{0}\cdot E_{1}+\Delta \mu H_{0}\cdot H_{1}\right)dv}{\int_{V_{0}}^{\;}\left(\epsilon_{0}{\left|E_{0}\right|}^{2}+\mu_{0}{\left|H_{0}\right|}^{2}\right)dv}$$

(2) $$\frac{Q_{0}-Q_{r}}{Q_{0}Q_{r}}=\frac{\int_{V_{0}}\left(\Delta \epsilon ʺE_{0}\cdot E_{1}+\Delta \mu ʺH_{0}\cdot H_{1}\right)dv}{\int_{V_{0}}^{\;}\left(\epsilon_{0}{\left|E_{0}\right|}^{2}+\mu_{0}{\left|H_{0}\right|}^{2}\right)dv}$$

with *f_r_*, *f*_0_, *Q_r_* and *Q*_0_ being the resonance frequency and Q-factor of the perturbed and unperturbed cavities, respectively. ***E***_1_ (***H***_1_) and ***E***_0_ (***H***_0_) represent the electric field (magnetic field) with and without perturbation, respectively. ∆*ϵ* and ∆*μ* denote the changes in permittivity and permeability when LUT is introduced, and *V*_0_ is the cavity volume. Equations (1) and (2) indicate that once the resonance frequency and Q-factor are specified, the complex dielectric constant of LUT can be accurately extracted.

### 2.2. Equivalent Circuit Model of Re-Entrant Cavity

**Figure 2:**
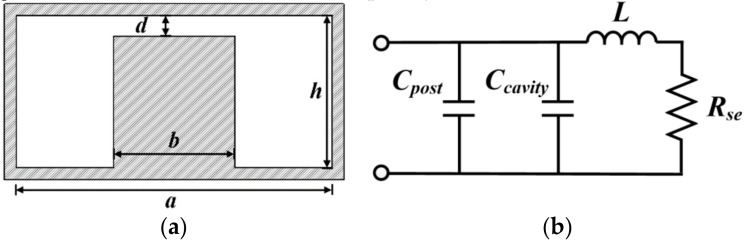
(**a**) Cross section of the square re-entrant cavity and (**b**) its equivalent circuit.

Figure 2a shows the cross section of a typical square re-entrant cavity. To obtain an accurate evaluation of resonant properties (i.e., resonance frequency and Q-factor) for the dimensioned cavity, an equivalent circuit model is established [29], as shown in Figure 2b. *C*_post_ is the gap capacitance formed between the post region and top lid of the cavity, while *C*_cavity_ is the remaining discontinuity capacitance of the cavity. *L* represents the total equivalent inductance and *R*_se_ is the equivalent series resistance which contains information of total losses throughout the cavity. By treating the cavity as a square shaped coaxial line terminated by a capacitive gap, these circuit parameters can be approximately computed by the following equations [29]:

(3) $$L=\frac{\mu_{0}h}{2\pi }ln\frac{a}{b}$$

(4) $$C_{\mathrm{cavity}}=\frac{4\epsilon_{0}b}{\sqrt{\pi }}ln\frac{e\sqrt{\frac{1}{\pi }{\left(a-b\right)}^{2}+h^{2}}}{2d}$$

(5) $$R_{\mathrm{se}}=\frac{1}{2\pi \delta \sigma }\left(\frac{\sqrt{\pi }\left(h-d\right)}{b}+\frac{\sqrt{\pi }h}{a}+2ln\frac{a}{b}\right)$$

where δ represents the skin depth and can be calculated as $\delta =\sqrt{\frac{2}{\omega \mu_{0}\sigma }}$, σ is the conductivity of conductor (e.g., 5.8 × 10^7^ S/m for copper). In the case of a highly loaded cavity (*d* $\ll \lambda_{0}$), the electric field between the capacitive post and the top lid is close to be uniform, and thus the gap capacitance *C*_post_ can be calculated using the standard formula for parallel–plate capacitor as:

(6) $$C_{\mathrm{post}}=\frac{\epsilon_{0}b^{2}}{d}$$

The resonance frequency and unloaded quality factor are finally obtained as follows [29,30,31]:

(7) $$f_{r}=\frac{1}{2\pi \sqrt{L\left(C_{\mathrm{cavity}}+C_{\mathrm{post}}\right)}}$$

(8) $$Q_{\mathrm{unloaded}}=\frac{\omega L}{R_{\mathrm{se}}}$$

With these formulas above, we can easily design a re-entrant cavity resonator operating at an arbitrary frequency. On the other hand, it also demonstrates that the changes in permittivity and dielectric loss inside the cavity would effectively alter the equivalent capacitance and resistance, thereby leading to the variations in resonance frequency and Q-factor.

## 3. Sensor Design

The sensor design in this work consists of two parts, namely the SIW re-entrant cavity resonator design and the microfluidic subsystem design. The primary objective for the cavity design is to obtain a compact size as well as a high Q-factor, while for microfluidic subsystem, it is to achieve a high sensitivity. By carefully incorporating these two parts together, a miniaturized and highly sensitive sensor with high accuracy can be realized. The detailed design processes are presented below.

### 3.1. Square SIW Re-Entrant Cavity Resonator Design

**Figure 3:**
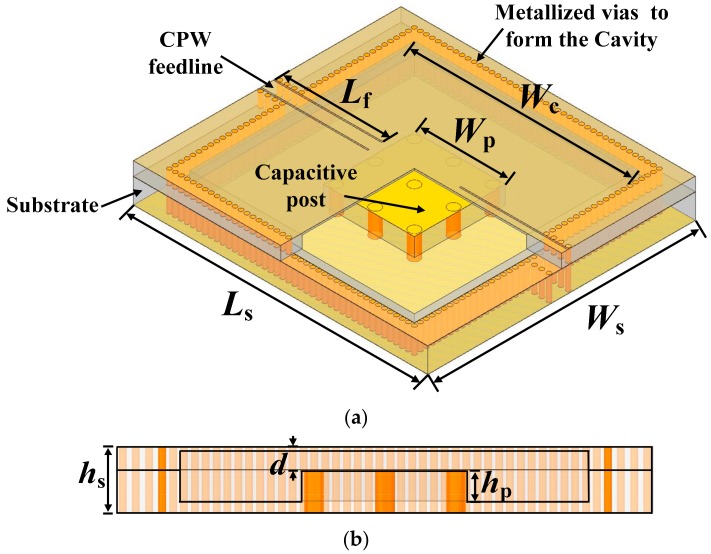
Geometric structure of the designed square SIW re-entrant cavity resonator: (**a**) 3-D view; (**b**) side view.

Following the equivalent circuit analyses in Section 2.2, a square SIW re-entrant cavity resonator operating in L band is designed, and its geometric structure is illustrated in Figure 3. The substrate is Rogers 4003 with a dielectric constant of 3.55 and a loss tangent of 0.0027. Two 35 μm copper films on both sides of the substrate are served as the top and bottom layers of the cavity, respectively. The ambient metallized vias, connecting the top and bottom copper films, are embedded in the substrate to form the four sidewalls of the cavity. In order to minimize the radiation losses between adjacent vias, the radius of vias *r_vc_* and the space between two adjacent vias *s_vc_* should comply with the following conditions [32]:

(9) $$\frac{s_{vc}}{4}\leq r_{vc}<\frac{\lambda_{g}}{10}$$

where $\lambda_{g}$ represent the guided wavelength. The capacitive post is realized through a square copper plate and a metallized via array. Two 50-Ω embedded coplanar waveguide (CPW) feedlines are used to excite the cavity. Optimized geometric parameters of the designed resonator are summarized in Table 1. Compared with conventional rectangular waveguide cavity, the designed resonator has achieved a great size reduction due to the large equivalent capacitance introduced by the capacitive post. For instance, a TE_101_ mode dominated conventional empty rectangular waveguide cavity operating at 2.19 GHz would generally occupy the dimensions of 112.45 mm × 86.36 mm × 43.18 mm (closed standard waveguide WR-340 with a length of $\lambda_{g}/2$) [27,33], while the resonator designed here only needs the sizes of 55 mm × 50 mm × 5.626 mm. Additionally, the planar configuration of our resonator, realized by SIW technology, also enables it to integrate with other planar circuits readily.

**Figure 4:**
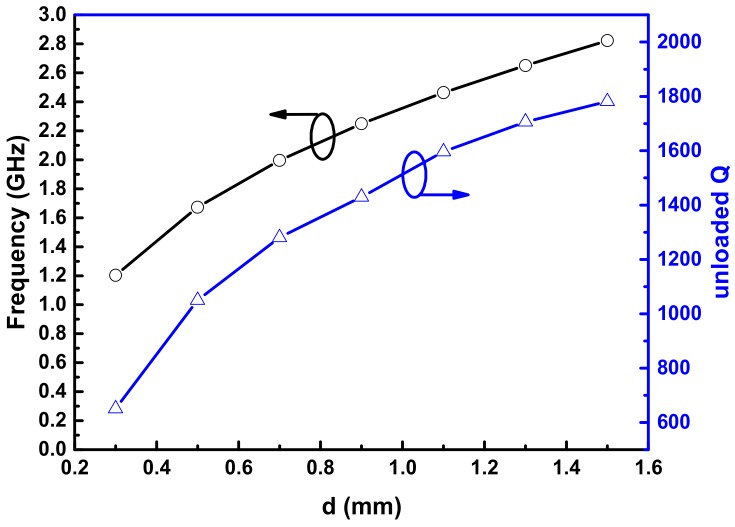
Simulated resonance frequency and unloaded Q-factor of the designed resonator versus gap spacing *d*.

According to Equations (3)–(8), the gap spacing *d* between the capacitive post and top lid of the cavity is a key parameter which can affect the resonance frequency and Q-factor of the resonator. To determine its value, the dependences of resonance frequency and Q-factor on *d* are simulated, and the corresponding results are plotted in Figure 4. The simulations are performed using high frequency structure simulator (HFSS). It is observed that with the increase of *d*, the unloaded Q-factor become higher and can reach the value of 1800. Nevertheless, the increasing *d* also leads to a higher resonance frequency, which means that the relative size of the resonator is enlarged. Therefore, taking both Q-factor and relative size of the resonator into consideration, the gap spacing *d* is carefully optimized to obtain a miniaturized resonant structure with a relatively high Q-factor.

### 3.2. Microfluidic Subsystem Design

**Figure 5:**
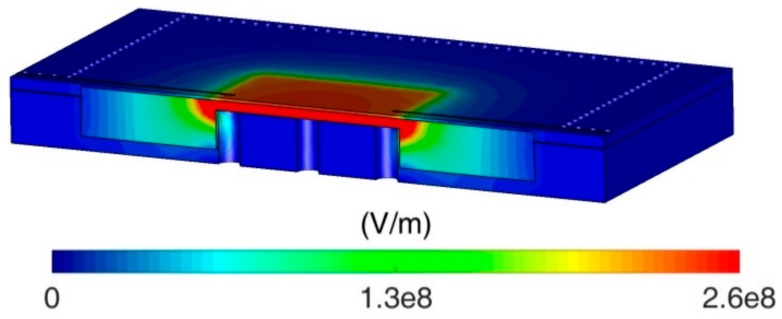
Electric field distribution of the cavity at resonance.

**Figure 6:**
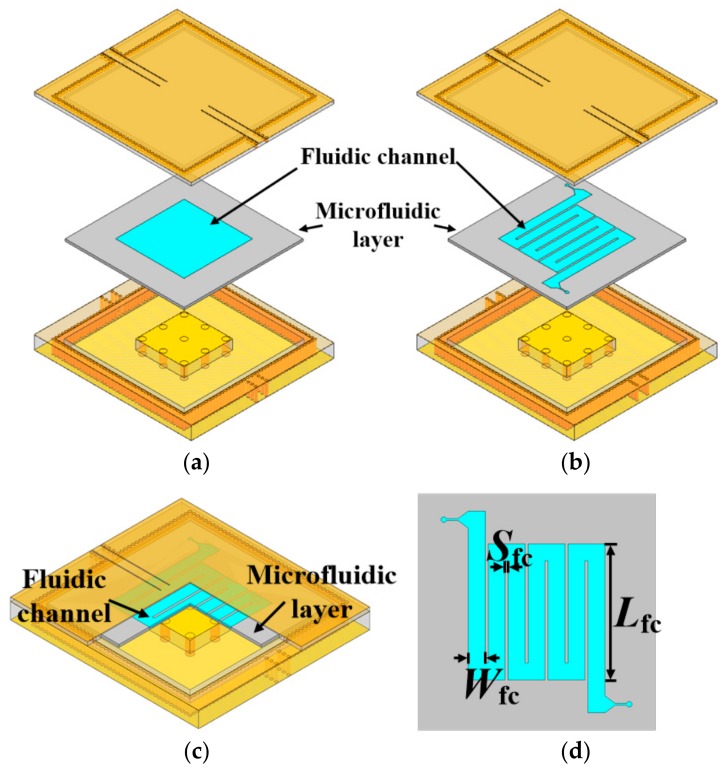
Diagram of the designed microfluidic subsystem: (**a**) 3-D view of the square plate channel design; (**b**,**c**) 3-D view of the winding channel design; (**d**) Front view of the winding channel design.

To achieve a larger perturbation and thus a higher sensitivity, the LUT is supposed to be placed at the region where electric field intensity is maximum. Figure 5 illustrates the electric field distribution of the designed cavity resonator, and a highly concentrated electric field located between the capacitive post and top lid of the cavity is clearly observed. According to this field distribution, the first type of microfluidic is designed, as depicted in Figure 6a. The microfluidic layer is placed between the post and the cavity lid, and its upper surface is bonded to the cavity to prevent the leakage of LUT. The fluidic channel is designed as a square plate covering the entire post region. This type of microfluidic can maximize the perturbation and achieve the theoretical highest sensitivity. Nevertheless, such a fluidic channel design is impractical because it would cause the problem of air bubbles or uneven filling when LUT is injected [10]. As an attempt to address this problem, another type of fluidic channel is proposed (see Figure 6b–d). In this design, the fluidic channel is modified as a winding shape so that LUT can be evenly filled in the channel, while the high sensitivity is preserved.

Polytetrafluoroethylene (PTFE) is chosen as the material of microfluidic layer due to its low cost and easy fabrication. Its relatively low dielectric loss, comparing with other frequently used material such as polydimethylsiloxane (PDMS) or polymethyl methacrylate (PMMA), is also beneficial for maintaining high Q-factor of the sensor. More importantly, it offers an excellent chemical resistance that enables the designed sensor to measure various organic solvents. However, there is an issue that the bond strength between PTFE microfluidic layer and cavity is relatively low, and thus the microfluidic layer may come off the cavity when inject pressure is too large, resulting in the leakage of LUT.

In this work, this problem is solved by designing a relatively wide and deep fluidic channel. According to the Young-Laplace equation, the minimum pressure required to induce flow is determined by the width (*W*_fc_) and depth (*H*_fc_) of microfluidic channel, which can be expressed as [34]:

(10) $$P_{c}\approx \frac{1}{W_{\mathrm{fc}}}+\frac{1}{H_{\mathrm{fc}}}$$

This suggests that the required minimum pressure *P_c_* can be decreased to the bearable range of PTFE by appropriately widening and deepening the fluidic channel. Final dimensions of the microfluidic are optimized as follows: *L*_fc_ = 24 mm, *W*_fc_ = 2.99 mm, *S*_fc_ = 0.5 mm. The thickness of the microfluidic layer is 0.8 mm, and the depth (*H*_fc_) of the fluidic channel is 0.45 mm.

## 4. Complex Permittivity Predictive Model Setup

**Figure 7:**
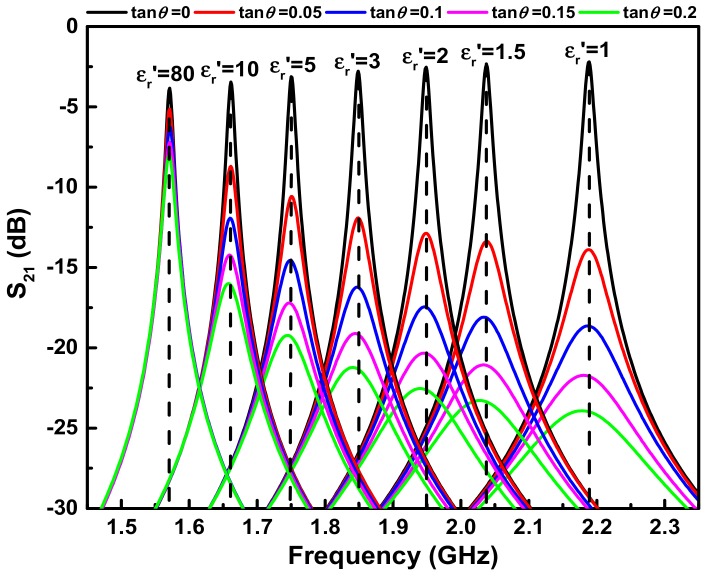
Simulated transmission response of the designed sensor with respect to the permittivity $\varepsilon_{r}^{\prime }$ and tan θ of LUT.

**Figure 8:**
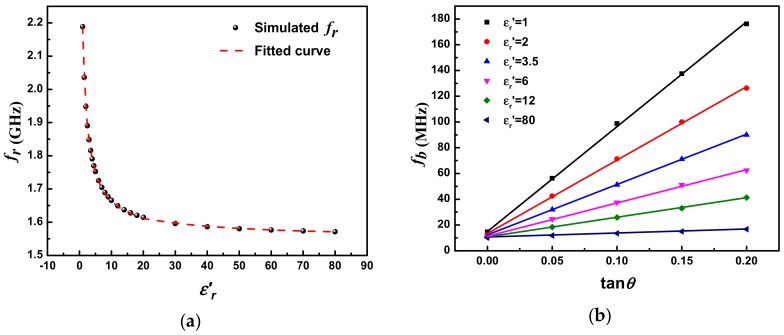
Relationship between (**a**) resonance frequency *f_r_* and permittivity $\varepsilon_{r}^{\prime }$ of LUT and (**b**) 3-dB bandwidth *f_b_* and tan θ of LUT.

Equations (1) and (2) in Section 2.1 give the quantitative relationships between the resonant characteristics and the dielectric properties of LUT. However, they are not suitable for extracting the complex permittivity of LUT due to their high complexity. In order to establish a simpler mathematical predictive model for quick and accurate characterization of LUT, numerical simulations of the sensor are carried out using HFSS. Figure 7 presents the simulated transmission responses of the designed sensor with various permittivities ($\varepsilon_{r}^{\prime }$) and loss tangents (tan θ) of LUT. It is apparent that a sharp resonant peak is generated at 2.19 GHz when the fluidic channel is empty (i.e., the case of $\varepsilon_{r}^{\prime }$ = 1 and tan θ = 0). The corresponding unloaded Q-factor is calculated as 673. The resonant peak shifts towards lower frequencies with the increase of $\varepsilon_{r}^{\prime }$, as would be expected. The maximum resonance frequency shift reaches 617.5 MHz when $\varepsilon_{r}^{\prime }$ varies from 1 to 80. Such a large frequency shift, equating to a high sensitivity, is highly desirable for the sensor because it is beneficial for improving accuracy. In addition, when $\varepsilon_{r}^{\prime }$ is fixed, the resonance frequency keeps almost unchanged with the increase of tan θ (in the range of tan θ < 0.2). To better characterize the connection between the resonance frequency and permittivity $\varepsilon_{r}^{\prime }$, the resonance frequency with varying $\varepsilon_{r}^{\prime }$ is simulated and plotted in Figure 8a. It can be found that the resonance frequency is related to $\varepsilon_{r}^{\prime }$ as an inverse proportional function. By using nonlinear curve fitting methods, the resonance frequency associating with the permittivity $\varepsilon_{r}^{\prime }$ of LUT can be given as:

(11) $$f_{r}={\left(\frac{100}{1.553\varepsilon_{r}^{\prime }-0.38825}+13.3903\right)}^{0.1703}$$

The same method is employed for the determination of loss tangent of LUT. In this work, 3-dB bandwidth *f_b_* is chosen as signature to extract tan θ due to the linear relationship between them. Figure 8b plots the simulated 3-dB bandwidth of the sensor as a function of tan θ at various $\varepsilon_{r}^{\prime }$. As can be seen, the 3-dB bandwidth *f_b_* increases linearly with the increase of tan θ when $\varepsilon_{r}^{\prime }$ is fixed. Oppositely, the slope of linear function *f_b_* (tan θ) tends to drop as $\varepsilon_{r}^{\prime }$ increases. The dependence of the slope on $\varepsilon_{r}^{\prime }$ is then analyzed and it is given as:

(12) $$k=1620.701{\left(\varepsilon_{r}^{\prime }+1.0889\right)}^{-0.92089}$$

Substituting Equation (12) to the linear function *f_b_* (tan θ) and fitting with the data in Figure 8b, we can finally obtain:

(13) $$f_{b}=1620.701{\left(\varepsilon_{r}^{\prime }+1.0889\right)}^{-0.92089}\tan\theta +12.6$$

**Figure 9:**
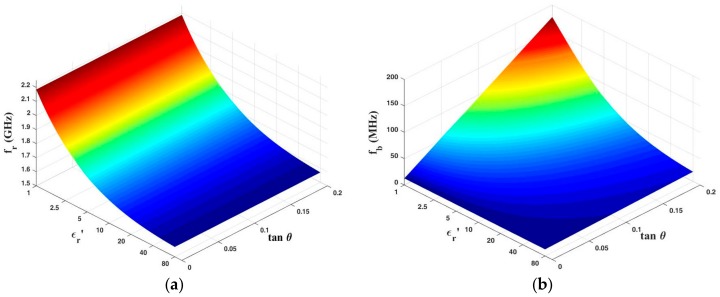
3D plots of the simulated (**a**) resonance frequency and (**b**) 3-dB bandwidth as a function of permittivity $\varepsilon_{r}^{\prime }$ and tan θ.

Equations (11) and (13) describe the quantitative dependences of the resonant properties on complex permittivity of LUT. For an unknown liquid, its corresponding *f_r_* and *f_b_* can be measured using the proposed sensor, and then the dielectric constant of the liquid can be determined according to Equation (11). Based on the known $\varepsilon_{r}^{\prime }$ and measured *f_b_*, the tan θ of the liquid can then be easily extracted using Equation (13). Finally, the imaginary part of permittivity can be calculated by $\varepsilon_{r}^{ʺ}$=$\varepsilon_{r}^{\prime }$∙tan θ. Figure 9a,b present the more visualized and comprehensive 3D-plots of *f_r_* and *f_b_* as a function of $\varepsilon_{r}^{\prime }$ and tan θ, respectively. Using this predictive model, the complex permittivity of LUT can be extracted quickly and accurately.

## 5. Fabrication and Measurement

### 5.1. Sensor Fabrication and Measurement Setup

**Figure 10:**
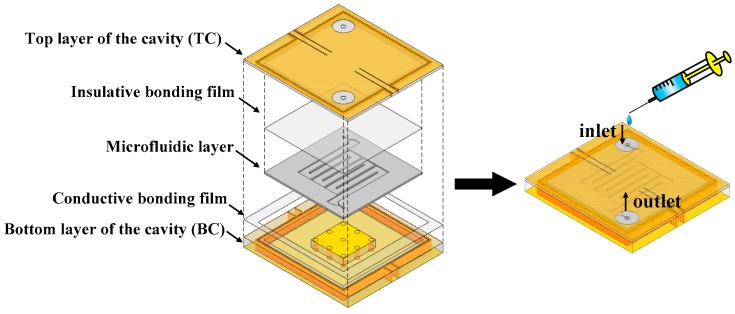
Assembly process of the proposed sensor.

**Figure 11:**
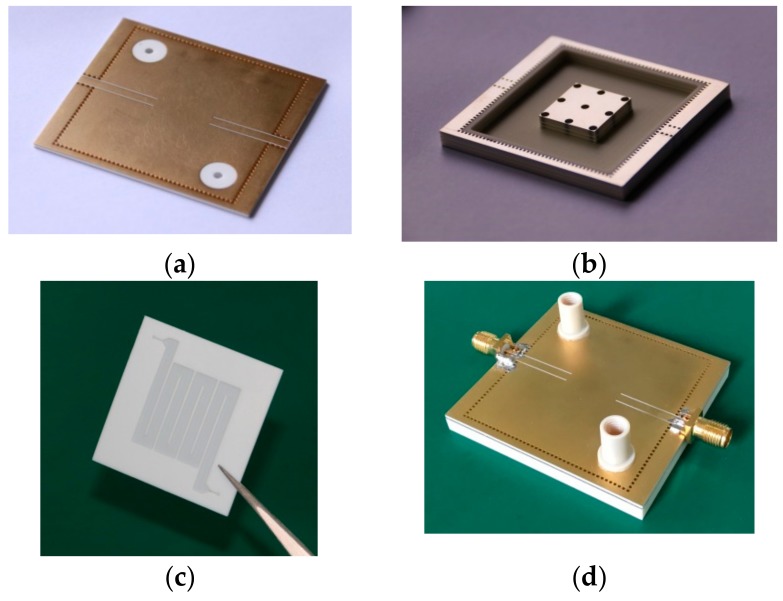
Photographs of the fabricated sensor: (**a**) the top layer; (**b**) the bottom layer of the cavity; (**c**) the microfluidic layer and (**d**) the assembled sensor.

The proposed sensor, consisting of the square SIW re-entrant cavity and the microfluidic subsystem, is fabricated using standard printed circuit board (PCB) technology and micromachining processes. Its assembling process is illustrated in Figure 10. From the structure diagram, the re-entrant cavity is divided into two parts, the top layer (TC) and bottom layer (BC). A square groove, with the same dimensions of the microfluidic layer, is etched in the lower surface of TC. Then, the microfluidic layer is embedded in the groove, and is bonded to TC using an insulated bonding film in order to avoid the leakage of LUT. In the upper surface of TC, two via holes with radius of 1 mm are introduced for the injection and extraction of LUT. To build the capacitive post, an annular groove with a depth of 3.372 mm is dug on BC using micromachining process. Finally, the TC and BC is glued together using a conductive bonding film. Figure 11a–d shows the photographs of the fabricated TC, BC, microfluidic layer and the assembled sensor prototype, respectively.

**Figure 12:**
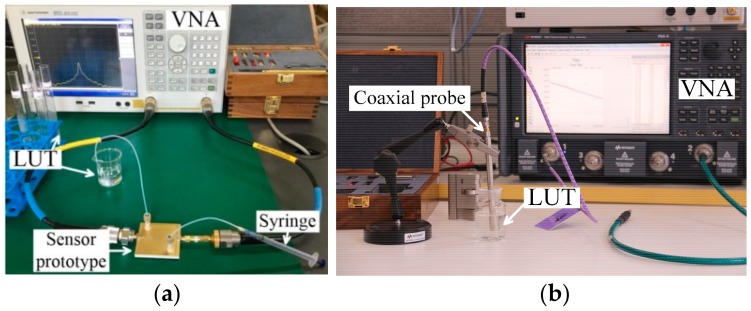
The measurement setup of (**a**) the proposed sensor in this work and (**b**) the Keysight coaxial probe kit (N1500A).

The measurement setup for the proposed sensor is shown in Figure 12a. The liquids to be measured are injected into fluidic channel using a syringe. Note that the inject rate should be relatively slow and keep constant, otherwise it may cause the problem of air bubbles or uneven filling, finally resulting in the inaccuracy of measured results.

The fabricated sensor prototype, filled with liquid sample, is connected to a vector network analyzer (E5071C, Agilent, Beijing, China) to obtain the required resonant properties. The vector network analyzer is calibrated using standard calibration kit 85052D before the measurements. As comparisons, the dielectric properties of liquid samples are also measured using coaxial probe kit (N1500A, Keysight, Chengdu, China), as shown in Figure 12b. All the measurements are performed at room temperature (25 °C).

### 5.2. Complex Permittivity Measurement of Pure Liquids

To validate the performance of the proposed sensor, four pure liquid samples [hexane, ethyl acetate, dimethyl sulfoxide (DMSO) and water] are measured and analyzed. These chemical samples, with the purity of A.R. grade, are purchased from Sinopharm Chemical Reagent Co., Ltd. (Shanghai, China). Each sample is slowly injected into the sensor until the fluidic channel is fully filled, and then the resonance frequencies and 3-dB bandwidths are recorded when the transmission responses remain stable. To improve the reliability of measured results, the measurement of each sample is repeated six times, and the final result is averaged from the six measurements. Since these four liquid samples exhibit different dielectric properties, any prior sample residual in fluidic channel would affect the accuracy of subsequent measurements. To minimize this effect, the fluidic channel is rinsed with deionized water and dried with hot air for 30 s after each kind of sample measurement is completed.

**Figure 13:**
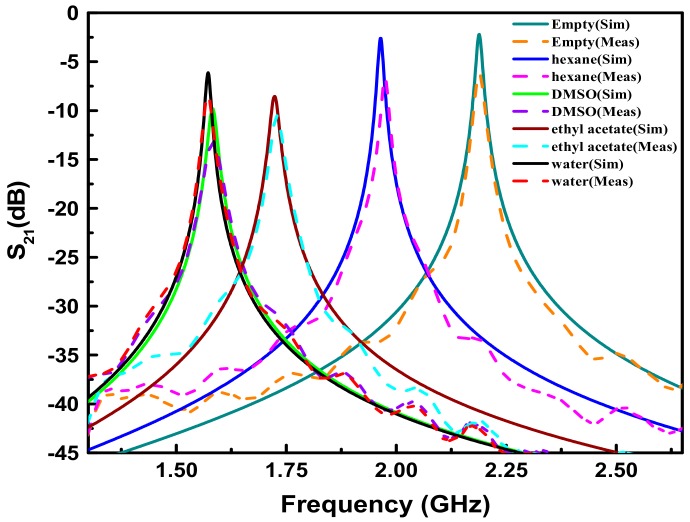
Simulated (solid lines) and measured (dash lines) transmission responses S_21_ of the proposed sensor for various liquids.

Figure 13 presents the simulated and measured transmission responses for various liquid samples. The complex permittivity of liquids used in simulations are calculated by the Debye relaxation equation [35,36]:

(14) $$\varepsilon_{r}^{\prime }-j\varepsilon_{r}^{ʺ}=\varepsilon_{\infty }+\frac{\varepsilon_{s}-\varepsilon_{\infty }}{1+j\omega \tau }$$

where $\varepsilon_{s}$ and $\varepsilon_{\infty }$ denote the static permittivity and the high-frequency permittivity limit, respectively. τ represents the relaxation time and can be computed as $\tau ={\left(2\pi f_{c}\right)}^{-1}$, with *f*_c_ being the relaxation frequency. The accurate values of these model parameters ($\varepsilon_{s}$, $\varepsilon_{\infty }$ and *f_c_*) for the chemicals used in this paper are taken from Ref. [35,36]. As shown in Figure 13, the measured resonance frequency and 3-dB bandwidth change significantly when the proposed sensor is filled by different mediums. The measured resonance frequency shift between air and water achieves 617.3 MHz, showing the high sensitivity of the sensor. In addition, all the measured results agree well with the simulated ones except a decrease in peak magnitude. The decrease in magnitude is mainly attributed to the losses from conductive bonding film, metallic vias and SMA connectors, and it would result in the increase of 3-dB bandwidth and finally lead to the discrepancies in measured dielectric properties of LUT. Consequently, the predictive model established in Section 4 needs to be modified according to the measured results, so as to eliminate these discrepancies. For this purpose, the simulated and measured transmission responses of empty channel are used as the references, and then the predictive model for extracting complex permittivity of LUT is modified as:

(15) $$\{\begin{array}{c} \;\varepsilon_{r}^{\prime }=\frac{100}{1.553f_{r}{}^{5.87}-20.795}+0.25\;\\ \varepsilon_{r}^{ʺ}=\frac{f_{b}-12.6}{8427.6452{\left(\varepsilon_{r}^{\prime }+1.0889\right)}^{-0.92089}}\times \varepsilon_{r}^{\prime } \end{array}$$

Compared with Equations (11) and (13), Equation (15) given above is also more convenient for calculation. Table 2 summarizes the simulated and measured results (*f_r_* and *f_b_*) for various liquid samples, together with the comparison between the theoretical and measured complex permittivities of LUT. As can be seen, the maximum relative error between the measured complex permittivities using our sensor and the theoretical values obtained by Debye relaxation equations is only 3.24% (the case of hexane). It means that a highly accurate complex permittivity measurement of pure liquid is realized successfully using the proposed method. Moreover, the small standard deviations of multiple measured results (shown in the brackets of Table 2) also demonstrate the good repeatability of the sensor.

### 5.3. Binary Liquid Mixture Measurement

In this section, the mixtures of acetone and water are employed to verify the ability of the proposed sensor to characterize binary liquid mixtures. For this purpose, acetone/water mixtures with various concentrations (0%, 20%, 40%, 60%, 80% and 100%) are carefully prepared using a pipette. The bulk volume of each mixture is 10 mL. Note that a larger bulk volume can effectively reduce the errors caused in mixture preparation process. The theoretical complex permittivity of binary liquid mixtures with different concentrations can be computed by the dielectric mixture equation given below [25,37]:

(16) $$\varepsilon_{r}=\varepsilon_{1}\cdot \left[\frac{\left(2\varepsilon_{1}+\varepsilon_{2}\right)+2V_{f}\left(\varepsilon_{2}-\varepsilon_{1}\right)}{\left(2\varepsilon_{1}+\varepsilon_{2}\right)-V_{f}\left(\varepsilon_{2}-\varepsilon_{1}\right)}\right]$$

where $\varepsilon_{r}$, $\varepsilon_{1}$ and $\varepsilon_{2}$ are the complex permittivities of the binary mixture, host liquid (i.e., acetone in our case) and additive liquid (i.e., water in our case), respectively. *V_f_* is the volume fraction of water in the acetone/water mixtures. The complex permittivities of acetone and water are calculated using Equation (14).

**Figure 14:**
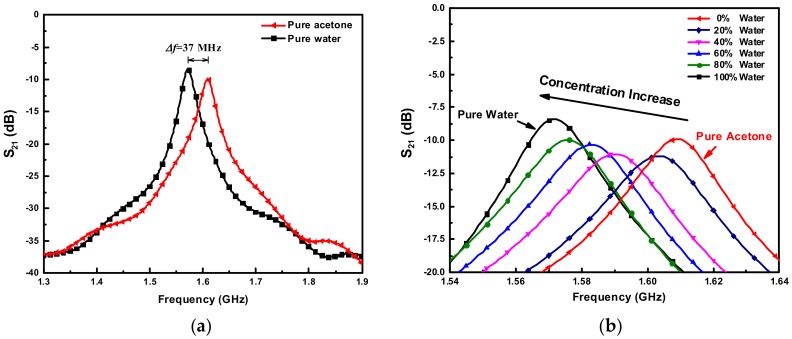
Measured transmission responses S_21_ of the proposed sensor for (**a**) pure water and acetone and (**b**) acetone/water mixtures in various concentrations.

**Figure 15:**
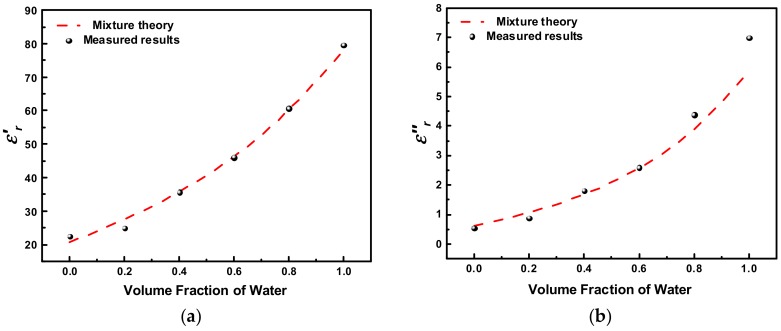
Theoretical and measured (**a**) $\varepsilon_{r}^{\prime }$ and (**b**) $\varepsilon_{r}^{ʺ}$ versus volume fraction of water.

The measured transmission responses of the proposed sensor for pure water, pure acetone, as well as the acetone/water mixtures in various concentrations are shown in Figure 14. There is a frequency gap of 37 MHz between the resonant peaks of pure water and acetone, and all the peaks of acetone/water mixtures are evenly distributed within this gap area. Each concentration of the mixture corresponds to a unique resonance frequency (with no overlapping). More specifically, the resonance frequency is monotonically reduced from 1.609 GHz to 1.572 GHz when the volume fraction of water increases from 0% to 100%. This is because the real part of complex permittivity of water is higher than that of acetone. Analogously, the 3-dB bandwidth also varies with volume fraction of water as a result of the different imaginary part of complex permittivity between water and acetone. With the measured resonance frequencies and 3-dB bandwidths, the complex permittivities of acetone/water mixtures in various concentrations are calculated using Equation (15) and plotted in Figure 15, where the corresponding theoretical values obtained from Equation (16) are also depicted. A good agreement between the measured results and the theoretical values can be observed. It demonstrates that the proposed sensor is also capable of accurately characterizing the complex permittivity of binary liquid mixtures.

**Figure 16:**
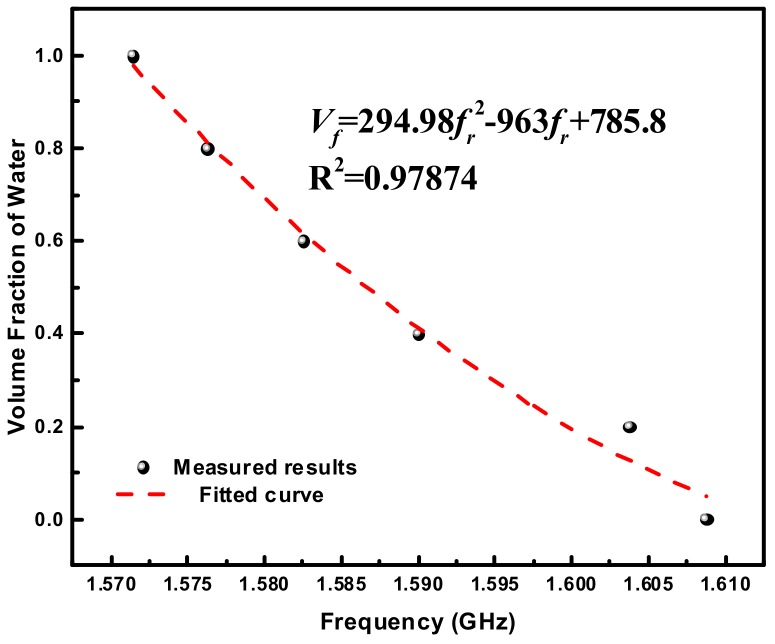
Plots of the volume fraction of water as a function of the resonance frequency.

Except for dielectric properties measurement, it is also meaningful to realize the quantification of a given binary liquid mixture [38,39]. Taking the acetone/water mixtures as an example, Figure 16 plots the volume fraction of water as a function of resonance frequency. By employing polynomial fitting methods, the connection between the volume fraction of water and the resonance frequency is given as:

(17) $$V_{f}=294.98f_{r}^{2}-963f_{r}+785.8$$

where *V_f_* is the volume fraction of water in acetone/water mixture, and *f_r_* is the measured resonance frequency. The coefficient of determination *R*^2^ is calculated to be 0.97874, indicating that Equation (17) fits well with the measured results. Based on Equation (17), we can easily quantify the concentration ratios of arbitrary given acetone/water mixtures. The same method is also suitable for other common solutions such as acetonitrile/toluene mixture, DMSO/water mixtures, etc.

## 6. Discussion

**Figure 17:**
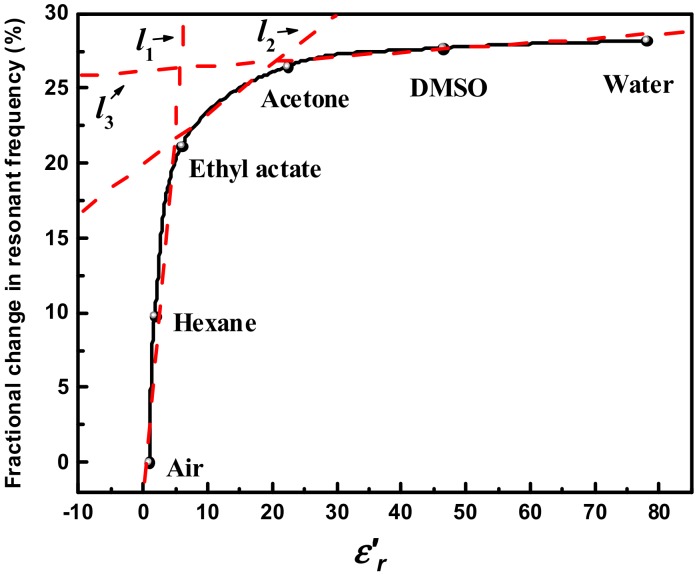
Plot of the sensitivity as a function of $\varepsilon_{r}^{\prime }$

Figure 17 presents the fractional changes in resonance frequency for the measured liquid samples. The proposed sensor exhibits an extremely high sensitivity when LUT has a relatively low dielectric constant ($1<\varepsilon_{r}^{\prime }<6$). This excellent sensitivity performance enables the proposed sensor to easily discriminate two liquids with very close dielectric properties or detect tiny changes of some biomacromolecules (e.g., alpha-fetoprotein in human serum). Oppositely, when $\varepsilon_{r}^{\prime }$ is higher than 20, the fractional changes in resonance frequency increase slowly with the increase of $\varepsilon_{r}^{\prime }$, corresponding to a relatively low sensitivity. This is because the sample polarizability is close to saturation within such high permittivity ranges, and thus the resonance frequency can be hardly changed with the dielectric constant [40]. In order to quantify the sensitivity of the designed sensor, the curve of fractional changes in resonance frequency is piecewise linearly approximated as three lines, which are *l*_1_ for $1<\varepsilon_{r}^{\prime }<6$, *l*_2_ for $6<\varepsilon_{r}^{\prime }<20$ and *l*_3_ for $\varepsilon_{r}^{\prime }>20$, respectively. The sensitivities in these three permittivity ranges, namely the slopes of these three lines, are then calculated to be 4.93%/$\varepsilon_{r}^{\prime }$, 0.33%/$\varepsilon_{r}^{\prime }$ and 0.034%/$\varepsilon_{r}^{\prime }$, respectively. It should be pointed out that despite the lower sensitivity when $\varepsilon_{r}^{\prime }>20$, the designed sensor is still competent to characterize the liquids with high dielectric constants. This is well demonstrated by the measurement of acetone/water mixtures in Section 5.3. Table 3 summarizes the comparisons between this work and other recently reported works in terms of some key figure-of-merits. For fairer comparison, the sensitivities of all these sensors are calculated using the following formula [41,42]:

(18) $$\mathrm{Sensitivity}=\frac{\Delta F}{\varepsilon_{r}^{\prime }\left(\mathrm{water}\right)-1}$$

where ΔF is the relative frequency shift between water and air, and is calculated as $\Delta F=\frac{f_{air}-f_{water}}{f_{air}}$. $\varepsilon_{r}^{\prime }$(water) is the permittivity of water at operating frequency. Compared with these state-of-art sensors listed in Table 3, the presented one in this work achieves a very competitive sensitivity, as well as a relatively compact size. The maximum error of only 3.24% also shows high accuracy of the proposed sensor. In fact, the errors are mainly caused by the fabrication tolerances and uncertainties in measurement environment, and they can be further reduced by more precise fabrications or using a mass of liquid samples with known permittivities to calibrate the sensor.

It should be noted that the presented sensor is more suitable for characterizing the complex permittivity of liquids with relatively low losses (tan θ < 0.2). The accuracy would be gradually deteriorated when the tan θ gets higher. This is due to the fact that for high loss tangents the linear relationship between 3-dB bandwidth and loss tangent (shown in Figure 8) no longer holds, and the influence of loss tangent on resonance frequency also becomes non-negligible. However, this limitation can be solved by means of reducing the effective volume of fluidic channel at the cost of sacrificing the sensitivity. In other words, the geometric structure of the proposed sensor can be flexibly adjusted to acquire a high sensitivity or a broad measurement range, depending on the practical requirements.

## 7. Conclusions

In this work, a novel microfluidic sensing concept based on square SIW re-entrant cavity for characterizing the complex permittivity of liquid is demonstrated both numerically and experimentally. A sensor prototype operating at 1.57–2.19 GHz with a dimension of 55 mm × 50 mm × 5.626 mm is designed and fabricated. It also can be applied to any other frequency band thanks to its high frequency tunability. By exploiting the intense electric field confined within the gap region of the cavity together with a modified winding microfluidic channel, a sensitivity up to 4.93%/$\varepsilon_{r}^{\prime }$ is successfully realized (in the case of $1<\varepsilon_{r}^{\prime }<6$). The introduction of microfluidic technology also makes the proposed sensor non-invasive, contactless and reusable. The maximum relative error between the measured permittivities of LUT and the theoretical values obtained by Debye relaxation equations is only 3.24%, showing the high accuracy of the presented method. Moreover, our sensor has also been proven to be capable of quantifying the concentration ratio of a given binary mixture. Due to the compact size, low cost, ease of integration, and most importantly, the prominent sensing performances, the proposed sensor may have potential applications in many areas such as biological, chemical, and pharmaceutical industry.

## Figures and Tables

**Table 1:** Geometric parameters of the designed resonator.

Parameter	Description	Value (mm)
*L_s_*	Substrate length	55
*W_s_*	Substrate width	50
*h_s_*	Substrate height	5.626
*L_f_*	CPW feedline length	20.2
*W_f_*	CPW feedline width	2.31
*W_cs_*	Width of the CPW slots	0.25
*W_c_*	Cavity width	42
*r_vc_*	Radius of via of the cavity	0.4
*s_vc_*	Space between two adjacent vias of the cavity	1.11
*W_p_*	Capacitive post width	17
*h_p_*	Capacitive post height	3.372
*r_vp_*	Radius of via of the capacitive post	1
*s_vp_*	Space between two adjacent vias of the capacitive post	7.3
*d*	Gap spacing between the capacitive post and top lid of the cavity	1.054

**Table 2:** Comparison between simulated and measured results.

Liquid Sample	Simulated *f_r_* (GHz)	Measured *f_r_* (Averaged)	Simulated *f_b_* (MHz)	Measured *f_b_* (Averaged)	Measured Complex Permittivity (Keysight Probe)	Theoretical Complex Permittivity [35,36]	Measured Complex Permittivity (Proposed Sensor)	Error (%)
Air	2.1888	2.18875	14.5	22.5	----	----	----	----
Hexane	1.9644	1.9735 (±0.0015)	13.1	19.58 (±3.5)	1.974 − j8.9*10^−3^	1.894 − j3.62*10^−4^	1.8325 − j4.1*10^−3^	3.24
Ethyl acetate	1.7237	1.7254 (±0.0023)	21.25	37.29 (±4.9)	6.88 − j0.159	5.918 − j0.1758	6 − j0.1069	1.38
DMSO	1.5825	1.5823 (±0.0015)	20	36.3 (±5.06)	43.4 − j8.1	45.1 − j7.327	46.4 − j4.57	2.88
Water	1.5721	1.5715 (±0.0005)	13.5	25.62 (±3.23)	79.15 − j6.1	77.98 − j5.828	79.52 − j6.99	1.97

**Table 3:** Comparison of other recently published works and this work.

Ref.	Resonator Type	Operating Frequency	$\mathrm{Sensitivity}\;(\%/\varepsilon_{r}^{\prime })$	Relative Size ^†^	Structure	Measurement Type	Maximum Error
[14]	Cylindrical dielectric resonator	10.5 GHz	0.0628	NA	Non-planar	Non-invasive and contactless	13.4%
[43]	SIW cavity resonator	8.96 GHz	0.0188	(0.46$\lambda_{0}\times$~×0.047$\lambda_{0}$) ^§^	Planar	Non-invasive and contactless	2.93%
[44]	Complementary SRR-loaded quarter mode SIW resonator	3.82 GHz	0.407	NR	Planar	Non-invasive and contactless	NR
[45]-1	Microstrip SRR	3.1035 GHz	0.026	0.37$\lambda_{0}\times$0.37$\lambda_{0}\times$0.016$\lambda_{0}$	Planar	Non-invasive and contactless	2.3%
[45]-2		3.1114 GHz	0.00655				2.9%
[46]	Coupled impedance resonator	1.37 GHz	0.13	(0.23$\lambda_{0}\times$~×0.002$\lambda_{0}$) ^†^	Planar	Non-invasive and contactless	NR
[47]	Coupled ring resonator	2.29 GHz	0.222	NR	Planar	Non-invasive and contactless	NR
[48]	SIW cavity resonator	17.08 GHz	0.16	1.7$\lambda_{0}\times$1.99$\lambda_{0}\times$0.147$\lambda_{0}$	Planar	Non-invasive and contactless	NR
[49]	SIW cavity resonator	5.8513 GHz	0.00683	1.46$\lambda_{0}\times$0.64$\lambda_{0}\times$0.019$\lambda_{0}$	Planar	Invasive	5%
This work	Square SIW re-entrant cavity resonator	2.18875 GHz	0.366	0.4$\lambda_{0}\times$0.36$\lambda_{0}\times$0.041$\lambda_{0}$	Planar	Non-invasive and contactless	3.24%

^†^$\lambda_{0}$ is the free space wavelength at operating frequency; ^§^ Length of the sensor is not mentioned in [43]; ^†^ The overall width of the sensor is not given in [46].
